# Management of Patulous Eustachian Tube

**DOI:** 10.31662/jmaj.2020-0007

**Published:** 2020-04-06

**Authors:** Ryoukichi Ikeda, Toshiaki Kikuchi, Hidetoshi Oshima, Toshimitsu Kobayashi

**Affiliations:** 1 Sen-En Rifu Otological Surgery Center, Miyagi, Japan; 2 Department of Otolaryngology-Head & Neck Surgery, Tohoku University School of Medicine, Sendai, Japan

**Keywords:** Eustachian tube, patulous Eustachian tube, surgical treatment, tympanic membrane, silicone plug

## Abstract

Patients with patulous Eustachian tubes (PET) suffer from annoying aural symptoms, such as voice or breath autophony, and aural fullness due to the ET’s abnormal patency. It may lead to an enormous reduction in quality of life. Various treatment methods, including conservative and surgical therapy, have been reported. In most cases, conservative treatment is sufficient to relieve patients of aural symptoms. However, some chronic and severe cases are resistant to traditional conservative therapy. Recently performed prospective and multicenter trials revealed the efficacy and safety of a silicone plug (Kobayashi plug) insertion for patients with severe PET. Patulous Eustachian tube handicap inventory-10 (PHI-10), tubal obstruction procedures, sitting computed tomography (CT), and ET function tests (tubo-tympano-aerodynamic graphy (TTAG) and sonotubometry) are useful for diagnosis as well as selecting candidates for surgery in severe cases.

## 1. Introduction

The Eustachian tube (ET) is the connection between the middle ear and nasopharyngeal space. It is closed under normal conditions and opens temporarily during swallowing ^(1), (2)^. The closed ET protects the middle ear from unwanted acoustic energy transmission from the nasopharynx, which is generated by self-vocalization and breathing. Patients with patulous ET (PET) suffer from annoying aural symptoms, such as aural fullness, voice, or breath autophony, due to the ET’s abnormal patency ^(3)^, reducing quality of life enormously. Various treatment methods have been reported ^(4), (5)^ but some controversy remains. In this review, we will describe treatments for this challenging disease.

## 2. Treatment of PET

### 2-1. Conservative treatment

In most cases, PET symptoms can be alleviated by patient explanation. This entails explaining the disease and mechanisms leading to symptom manifestation to the patient, providing reassurance, offering specific lifestyle guidance (rehydrating sufficiently during summer or after vigorous activity, avoiding weight loss, etc.), and prescribing conservative therapy ^(4)^. Self-instillation of physiological saline solution has been reported to be effective in controlling PET symptoms ^(6)^. Mucous-thickening agents ^(7)^ and topical irritants (e.g., Lugol-gel ^(8), (9)^, Bezold powder, or sodium iodide) to induce mucosal edema of the pharyngeal orifice can also provide temporary symptom relief.

Applying a paper patch to the tympanic membrane (TM) has also been proposed ^(10)^. A piece of steri strip (3M, Maplewood, USA), cut into a circular shape about 5 mm in diameter, was placed on the posterior-superior quadrant of the TM. It was reported to be effective in some PET cases where the primary symptom was aural fullness.

### 2-2. Surgical treatment

**Figure 1.**
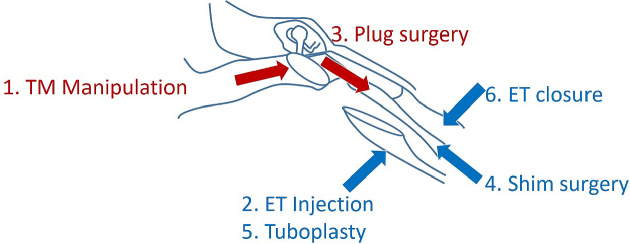
Surgical interventions classified by the procedure type: 1) tympanic membrane (TM) manipulation, 2) Eustachian tube (ET) injection, 3) plug surgery, 4) shim surgery, 5) tuboplasty, and 6) ET closure.

For chronic and severe cases resistant to conservative therapy, surgical procedures are considered. Surgical procedures are categorized by the procedure type, including TM manipulation (tympanostomy tube insertion and mass loading of the TM), plug surgery, ET injection, shim surgery, tuboplasty, and ET closure ^(4), (5)^. The former two methods are usually performed via a transtympanic approach, and the latter three treatments via a transnasal approach (Figure 1). A recent systematic review of PET surgical treatment revealed that plug surgery and shim surgery had shown relatively high efficacy and safety (unpublished data). The transtympanic silicone plug (Kobayashi Plug) insertion is categorized under plug surgery and is used for severe PET cases in Japan.

### 2-3. Kobayashi Plug

**Figure 2.**
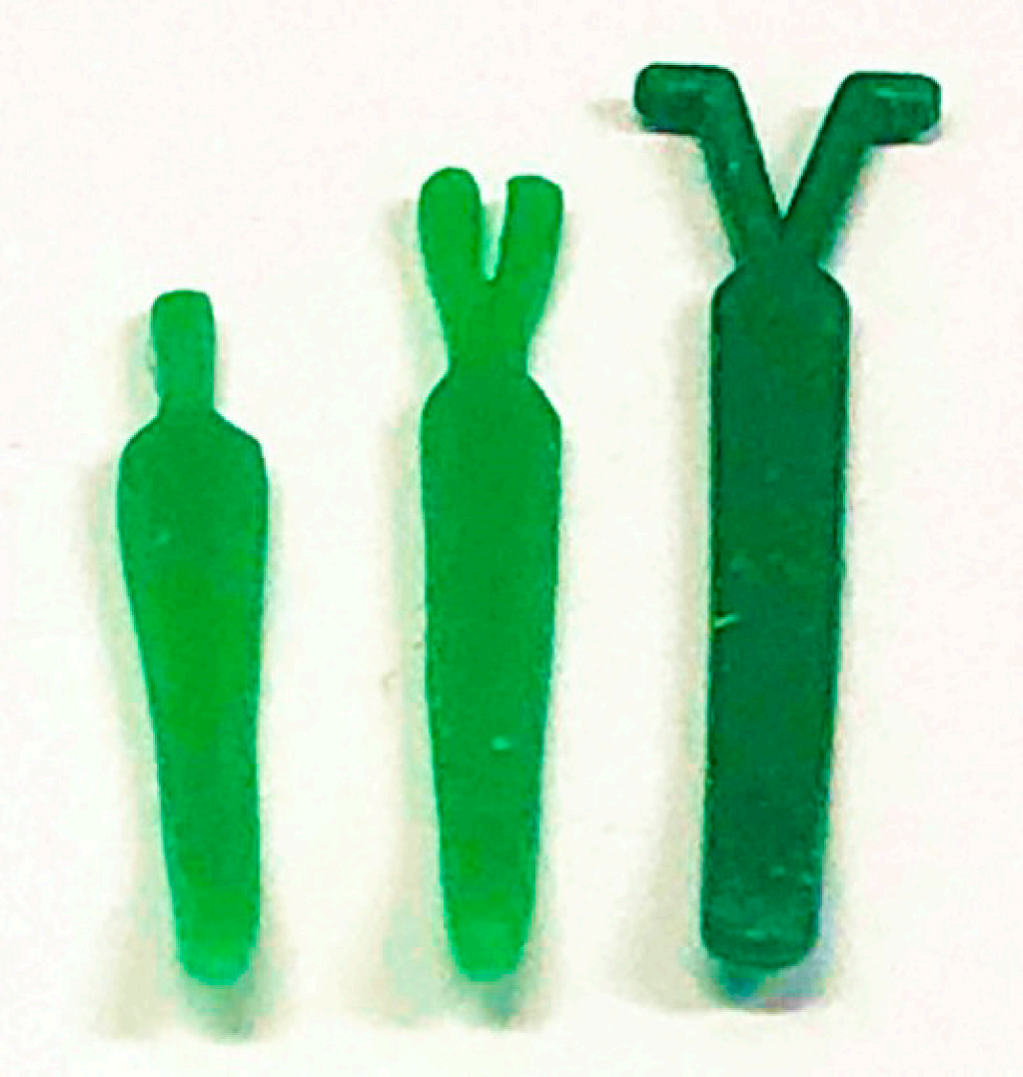
Kobayashi plug: Initial type (left), Prototype (middle), New type (right).

**Figure 3.**
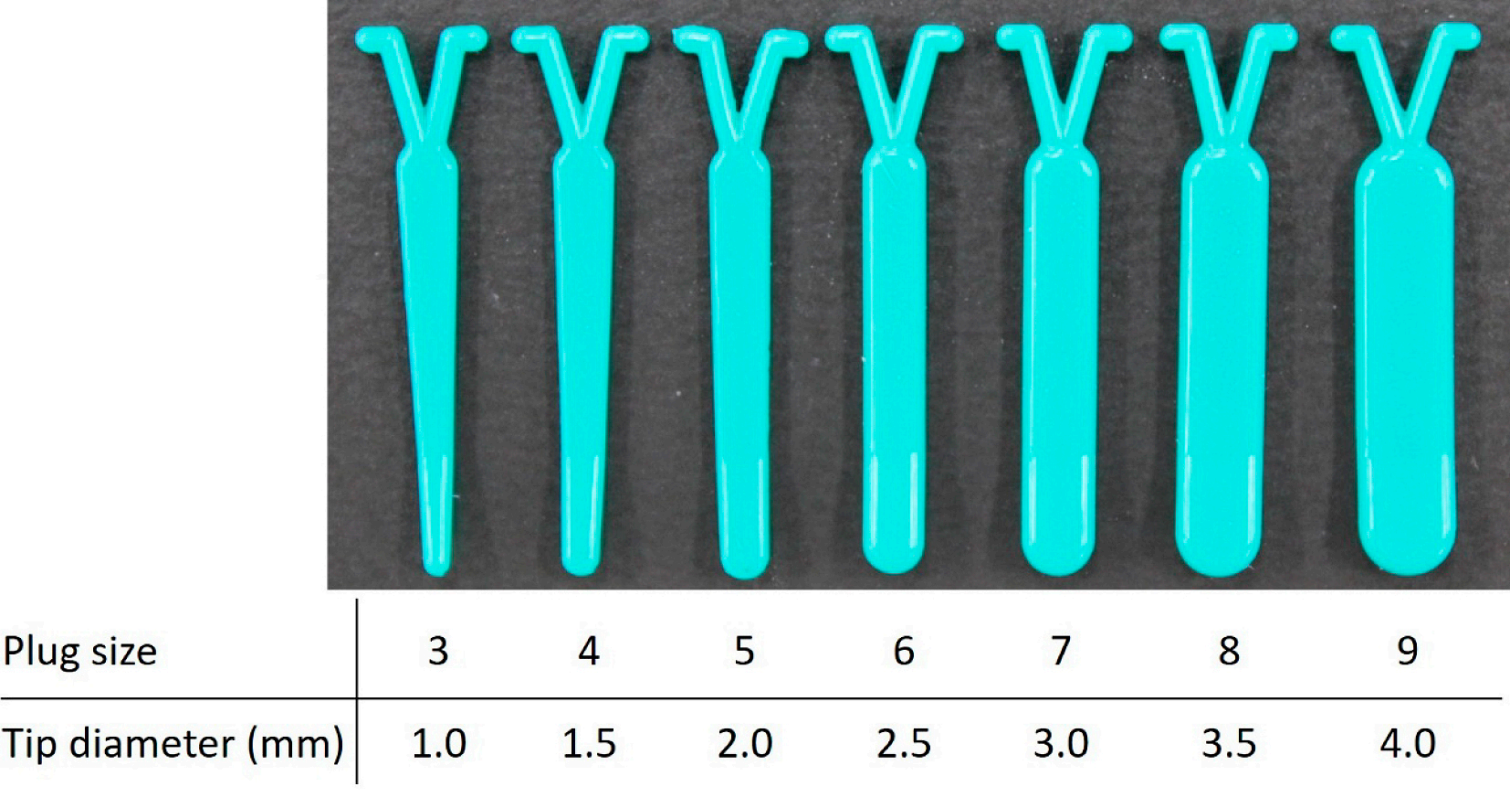
Kobayashi Plug Size (New Plug) between #3 and #9.

Bluestone et al. first described plugging the ET through the middle ear ^(11)^. They used an intravenous indwelling catheter; its lumen was filled with methyl methacrylate. The anterior tympanomeatal flap was raised, and a tympanostomy tube insertion was performed at the same time as plugging. We have developed a specially designed silicone plug (Kobayashi Plug) for treating patients with severe ^(9), (12), (13), (14), (15)^. The early prototype plug is smaller than the currently used type (Figure 2). The current type has a length of 23 mm and comes in different plug sizes, with differing tip diameters, to accommodate the ET variation in PET cases (type 3; 1.5mm to 9; 4.0mm) (Figure 3). The plug is slightly bent at the tip to facilitate insertion into the ET. A wing-like protrusion was designed for each tail of the plug to prevent descent into the nasopharynx, a problem frequently experienced by patients using the prototype plug, which lacked wing-like protrusions ^(13)^. This surgery is usually performed in cases of intractable “Definite PET” diagnosed by the JOS Diagnostic Criteria ^(16), (17)^ (Table 1) and resistant to at least six months of various conservative treatment methods ^(18)^. We have completed a prospective multicenter clinical trial in Japan supported by the development of New Drugs and Medical Devices (Japan Medical Association) from the Japan Agency for Medical Research and development, AMED.

**Table 1.** The Japan Otological Society Diagnostic Criteria for PET.

1. There are subjective symptoms
One or more of the following symptoms included: voice or breath autophony, and aural fullness
2. Tubal obstruction procedures (A or B) clearly improves symptoms
A. Posture change to the supine / lordotic positon
B. Pharyngeal orifice obstruction procedure (swab, gel, etc.)
3. There is at least one of the following objective findings of patent E-tube:
A. Respiratory fluctuation of the tympanic membrane
B. Variations of external auditory canal pressure synchronized with nasopharyngeal pressure
C. The sonotubometry shows (1) the probe tone sound pressure level is less than 100 dB or (2) an open plateau pattern.
Definite Patulous Eustachian tube (PET): 1 + 2 + 3
Possible Patulous Eustachian tube (PET) : 1+ (2 or 3)

#### 2-3-1. Surgical outcome

We previously reported 252 ears of 191 patients with PET who received a Kobayashi Plug insertion ^(13)^. The overall success rate was 83.0%. Furthermore, a prospective, multicenter trial was conducted for 30 patients with PET. The efficacy and safety of 28 and 27 patients, respectively, were analyzed. Twenty-three cases (82.1%) were considered successful ^(18)^.

#### 2-3-2. Complications

No severe or life-threatening complications have been observed. In a prospective, multicenter clinical trial (29 cases) ^(18)^, middle ear effusions (MEE), including transient effusions, occurred in 17.2% of cases. One case (3.4%) received a ventilation tube insertion for persistent MEE. TM perforation was found in 13.8%. No plug descent to the nasopharynx occurred. The hearing threshold (the pure-tone average of 0.5, 1, and 2 kHz of air-conduction) at the time of screening was 16.3±9 and at 3 and 6 months post-operatively were 18.6±10.9 dB HL and 18.6±13 dB HL, respectively, with no significant difference.

#### 2-3-3. Evaluation of ET for Kobayashi Plug surgery

##### Subjective findings

Subjective symptoms are evaluated by the patulous Eustachian tube handicap inventory-10 (PHI-10)^(19)^ (Table 2). The classification for grading severity is defined as 1) no handicap (0–8), 2) mild handicap (10–16), 3) moderate handicap (18–24), and 4) severe handicap (26–40). With respect to evaluation after surgery, no handicap (0–8) and mild handicap (10-16) are considered successful outcomes.

**Table 2.** Patulous Eustachian Tube Handicap Inventory-10 (PHI-10). 1) No Handicap (0–8). 2) Mild Handicap (10–16). 3) Moderate Handicap (18–24). 4) Severe Handicap (26–40).

No	Question	yes: 4	sometimes: 2	no: 0
1	Because of your symptom is it difficult for you to concentrate?			
2	Does the loudness of your symptom make it difficult for you to hear people?			
3	Does your symptom make you angry?			
4	Do you fell as though you cannot escape your symptom?			
5	Does your symptom interfere with your ability to enjoy social activities?			
6	Because of your symptom do you feel frustrated?			
7	Does your symptom interfere with your job or household responsibilities?			
8	Do you feel that your symptom has placed stress on your relationships with members of your family and friends?			
9	Do you find it difficult to focus your attention away from your symptom and on to other things?			
10	Does your symptom make you feel anxious?			

##### Tubal obstruction procedures

**Figure 4.**
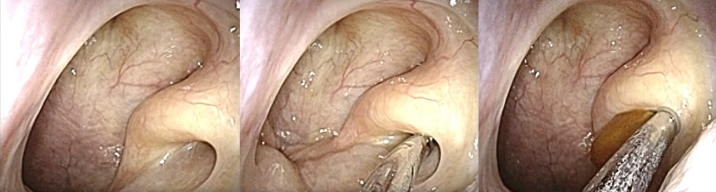
Lugol-gel injected into the Eustachian tube’s left pharyngeal orifice.

Before assessing improvement of symptoms after surgery, it should first be ascertained that the patient does indeed have “definite PET” by obstructing the ET's pharyngeal orifice. This occlusion is done transnasally using a swab, gel, etc., preoperatively (Figure 4), and if the patient’s aural symptoms do not improve after this maneuver, their symptoms are not due to PET. They may be due to other diseases, such as superior canal dehiscence syndrome sensorineural hearing loss, etc.

##### Sitting CT findings

**Figure 5.**
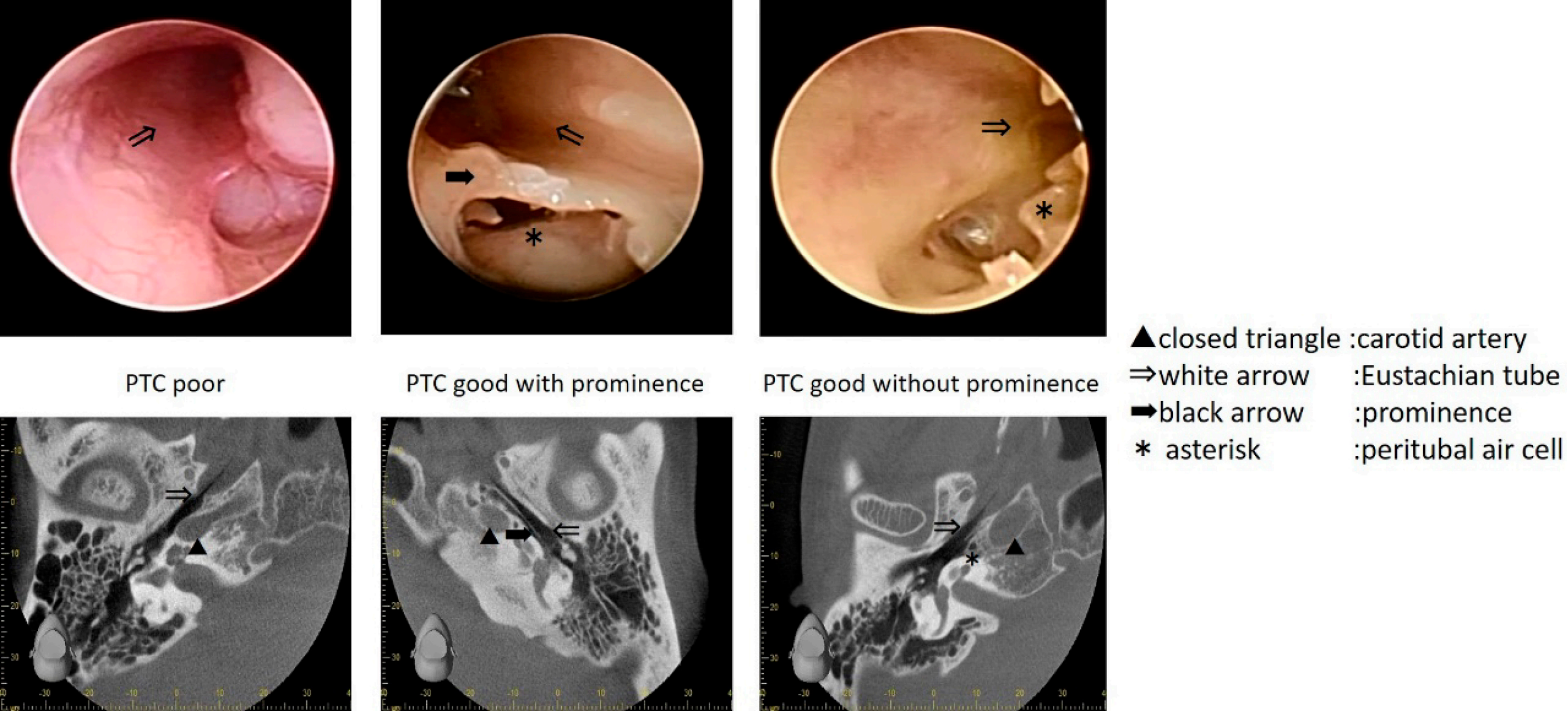
Endoscopic findings of the Eustachian tube’s bony portion (left peritubal cells (PTC) poor, middle PTC good with prominence, and right PTC good without prominence). White arrow; Eustachian tube, black arrow; prominence, and asterisk; peritubal air cell.

Sitting computed tomography (CT) is essential for preoperative ET evaluation by delineating the ET lumen’s shape throughout its course from the nasopharynx to the tympanic orifice ^(20), (21), (22), (23), (24)^. The anatomy of the ET’s tympanic portion is variable and categorized into three types, namely poor peritubal cell (PTC) types (Figure 5: left), good PTC types with prominence (Figure 5: middle), and good PTC types without prominence (Figure 5: right) ^(25)^. The good PTC type with prominence (Figure 5: middle) has a significantly smaller tympanic orifice width than the other types. The good PTC type, with prominence, has a false passage due to the ridge of bony prominence separating the ET’s tympanic portion into a lateral opening for the ET ^(26)^. Therefore, plug surgery is can be more difficult in this type than in the two other types.

##### ET function test

**Figure 6.**
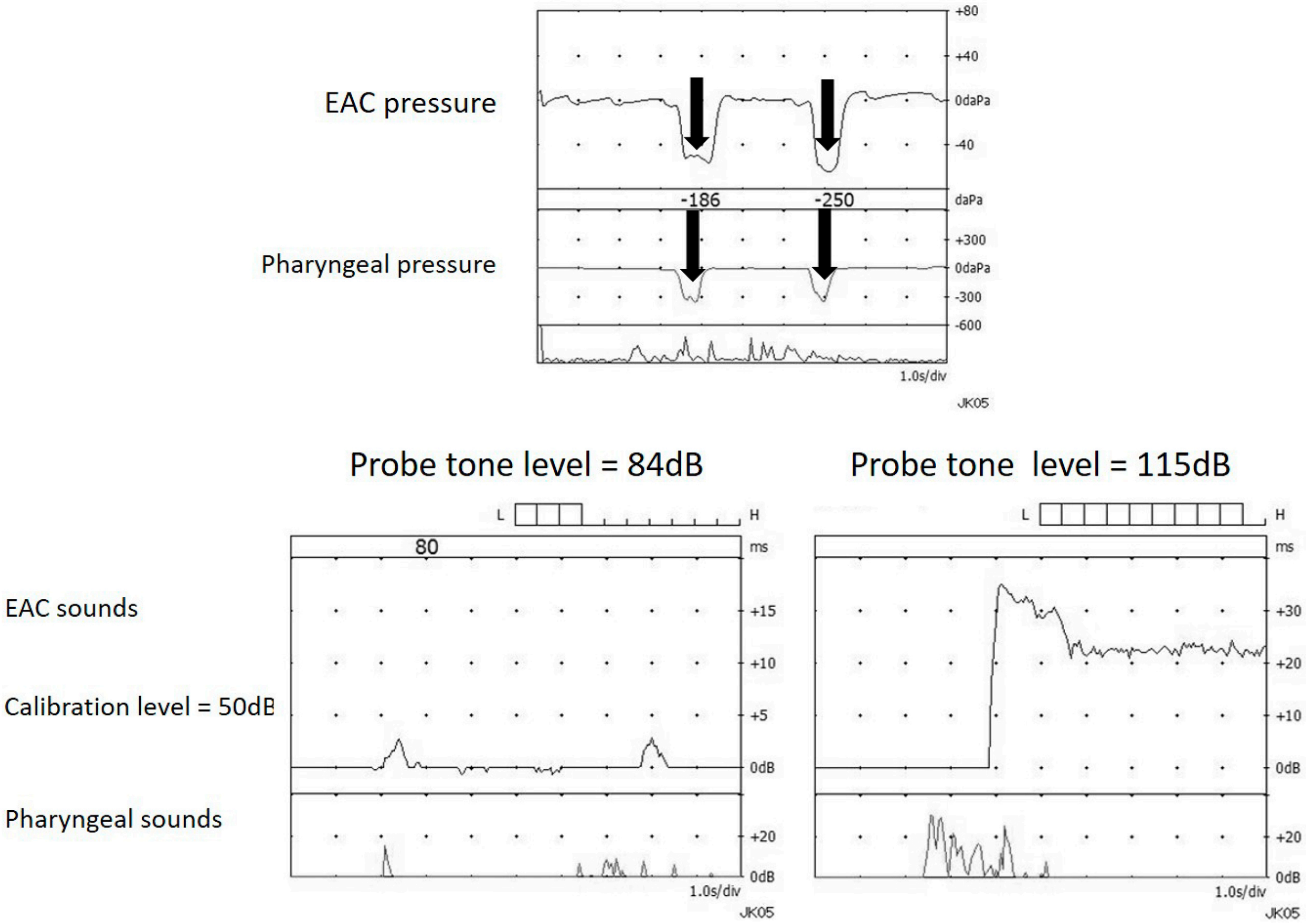
Typical tubo-tympano-aerodynamic graphy (TTAG) recordings in a Patulous Eustachian tube (PET) case (upper). In PET, synchronous changes in the external auditory canal (EAC) pressure are induced by respiration. Examples of typical sonotubometric recordings in PET cases (lower left and right). Lowering the probe tone SPL to less than 100dB (left). The ET opens when swallowing and remains continuously open thereafter (right).

ET function tests, such as tubo-tympano-aerodynamic graphy (TTAG) ^(27)^ and sonotubometry ^(28)^, were performed using a commercially available machine (JK05AD; Rion, Tokyo, Japan). Pressure changes in the external auditory canal (EAC) and the nasopharynx were coincidentally recorded using the TTAG’s manometry mode. Positive TTAG findings were determined as an EAC pressure change synchronous with that in the ipsilateral nasopharynx (Figure 6: upper). Sonotubometry automatically creates the input sound pressure level (SPL) at the nostril that enables a pre-set level of 50 dB SPL output in the EAC. Two findings were considered consistent with a patent ET, i.e. lowering of probe tone SPL to below 100 dB (Figure 6: lower left) or an “open plateau pattern” when the ET opens upon swallowing and remains open (Figure 6: lower right).

Sound transmission assessed by sonotubometry is more useful than pressure transmission assessed by TTAG in predicting PET’s morphological severity ^(29), (30)^. Thus, the sitting CT and sonotubometry are valuable tools to aid in plug size selection. Based on our previous study ^(31)^, we recommend the initial Kobayashi Plug size selection, based on preoperative probe tone SPL, using sonotubometry as follows: size #4; over 95dB, size #5; between 94 and 85 dB, size #6; lower than 84dB.

#### 2-3-4. Surgical steps

This surgery can be performed at either the outpatient clinic or in an operating room. We usually conducted this procedure in an operating room. 

**Figure 7.**
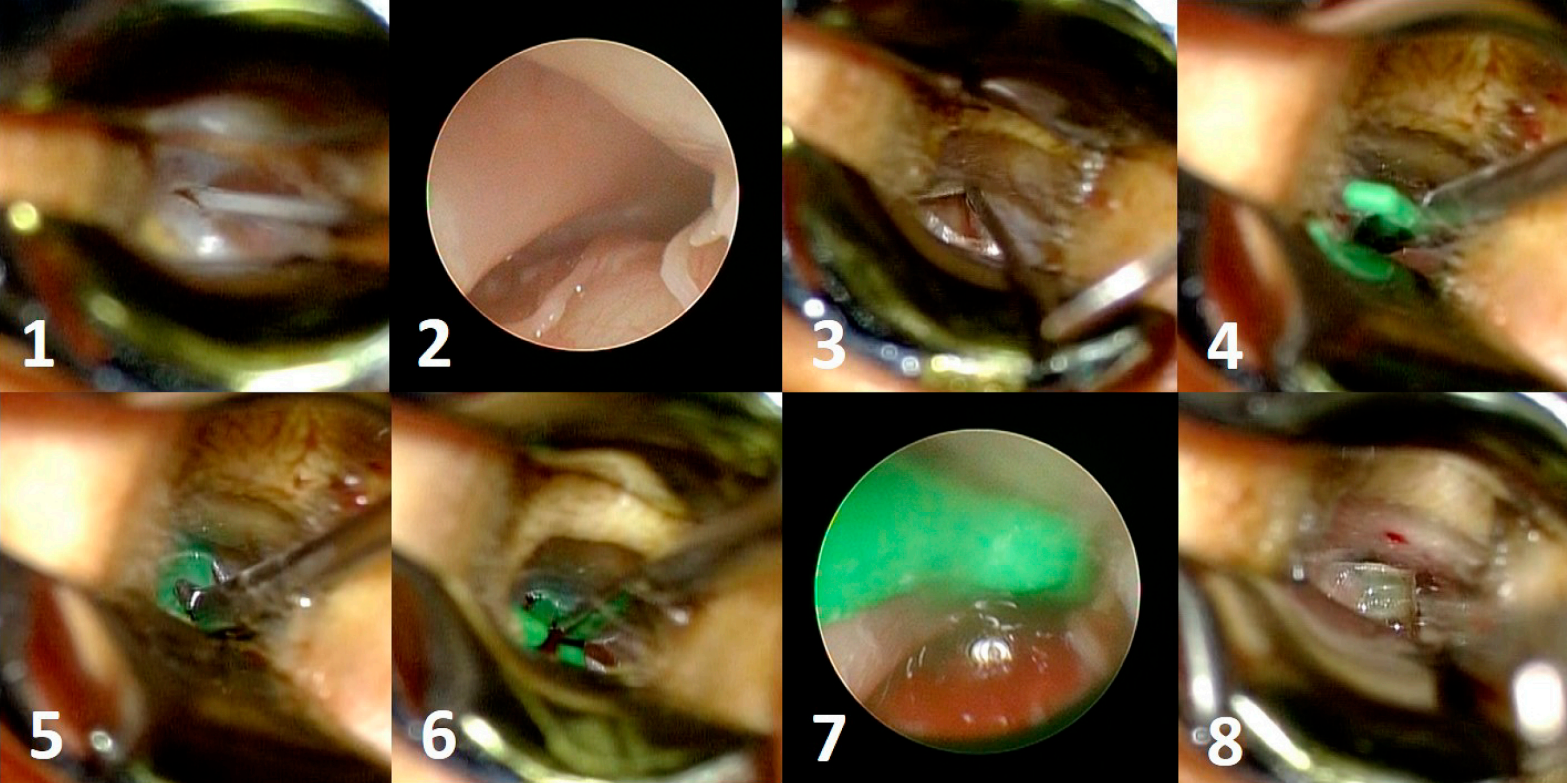
Surgical steps of Kobayashi plug insertion.

1. After draping, the EAC is anesthetized with an injection of 0.5–1.0 mL of 1% lidocaine at the EAC.

2. A small myringotomy (3 mm long) is performed in the TM’s anterosuperior quadrant (Figure 7-1).

3. The ET’s tympanic portion can be visualized directly via a myringotomy hole by 30-degree endoscopy with a 2.7mm diameter (Figure 7-2).

4. Local anesthesia (step 1) is not sufficient for analgesia in the ET’s bony and cartilage portion, so 0.05 ml of 4% lidocaine solution is sprayed onto the bony portion of the ET directly (Figure 7-3). Patients are advised to position themselves with the affected ear pointing straight up toward the ceiling. The posterior tympanic cavity is filled with a saline solution before spraying lidocaine to prevent vertigo due to inner ear anesthesia.

4. The plug body is made such that it is bent slightly toward the tip to facilitate insertion from a small myringotomy hole (Figure 7-4). After the tip is inserted, the plug is laid down toward the TM and inserted gently, sliding on the TM surface (Figure 7-5 and 6). If resistance is felt during insertion, the plug status should be checked via endoscopy. When a plug does not advance, in spite of being inserted into the ET, changing the plug to a smaller size should be considered. Adequate plug size is determined when slight resistance is felt. If no resistance is felt, the plug size may be too small.

5. The plug position is checked with 30-degree endoscopy inserted medial to the TM (Figure 7-7).

6. The middle ear is rinsed with 10 ml of the saline solution after inserting the plug.

7. A piece of paper is applied to patch the myringotomy hole (Figure 7-8).

## Conclusion

This review identifies and summarizes the existing evidence for conservative and surgical treatment of PET symptoms. Plug surgery, especially Kobayashi plug surgery, is effective and safe for severe PET cases. Moreover, multifaceted evaluation of the ET condition is essential to manage patients with PET.

## Article Information

### 

This article is based on the study, which received the Medical Research Encouragement Prize of The Japan Medical Association in 2019.

### Conflicts of Interest

None

### Sources of Funding

This work was supported by JSPS KAKENHI grant number 15K20175, 18K16872 and the Project Promoting Clinical Trials for Development of New Drugs and Medical Devices (Japan Medical Association) from Japan Agency for Medical Research and development, AMED grant number CCT-B-2806.
